# Interactions between acoustic challenges and processing depth in speech perception as measured by task-evoked pupil response

**DOI:** 10.3389/fpsyg.2022.959638

**Published:** 2022-10-25

**Authors:** Jing Shen, Laura P. Fitzgerald, Erin R. Kulick

**Affiliations:** ^1^Department of Communication Sciences and Disorders, College of Public Health, Temple University, Philadelphia, PA, United States; ^2^Department of Epidemiology and Biostatistics, College of Public Health, Temple University, Philadelphia, PA, United States

**Keywords:** speech perception, processing effort, task-evoked pupil response, processing depth, speech in noise

## Abstract

Speech perception under adverse conditions is a multistage process involving a dynamic interplay among acoustic, cognitive, and linguistic factors. Nevertheless, prior research has primarily focused on factors within this complex system in isolation. The primary goal of the present study was to examine the interaction between processing depth and the acoustic challenge of noise and its effect on processing effort during speech perception in noise. Two tasks were used to represent different depths of processing. The speech recognition task involved repeating back a sentence after auditory presentation (higher-level processing), while the tiredness judgment task entailed a subjective judgment of whether the speaker sounded tired (lower-level processing). The secondary goal of the study was to investigate whether pupil response to alteration of dynamic pitch cues stems from difficult linguistic processing of speech content in noise or a perceptual novelty effect due to the unnatural pitch contours. Task-evoked peak pupil response from two groups of younger adult participants with typical hearing was measured in two experiments. Both tasks (speech recognition and tiredness judgment) were implemented in both experiments, and stimuli were presented with background noise in Experiment 1 and without noise in Experiment 2. Increased peak pupil dilation was associated with deeper processing (i.e., the speech recognition task), particularly in the presence of background noise. Importantly, there is a non-additive interaction between noise and task, as demonstrated by the heightened peak pupil dilation to noise in the speech recognition task as compared to in the tiredness judgment task. Additionally, peak pupil dilation data suggest dynamic pitch alteration induced an increased perceptual novelty effect rather than reflecting effortful linguistic processing of the speech content in noise. These findings extend current theories of speech perception under adverse conditions by demonstrating that the level of processing effort expended by a listener is influenced by the interaction between acoustic challenges and depth of linguistic processing. The study also provides a foundation for future work to investigate the effects of this complex interaction in clinical populations who experience both hearing and cognitive challenges.

## Introduction

Speech perception is a multistage process that begins with the auditory reception of acoustic stimulation and culminates in the listener’s interpretation of the speaker’s message. The auditory and cognitive systems process acoustic input and interpret a speaker’s message within a matter of milliseconds (Rönnberg et al., 2013). When this process happens under adverse conditions, such as an environment with background noise, the auditory and cognitive systems must deploy strategies to navigate the difficulty and allow the listener to comprehend the meaning of the utterance. During speech perception in noise, for instance, the acoustic signals of the target speech must first be detected and perceptually separated from the acoustic stream (Shinn-Cunningham, 2008). In the recognition stage, linguistic operations at the word-level occur, including phonological analysis and word identification (Gordon-Salant et al., 2020). Any missing speech signal due to acoustic degradation from noise must also be resolved at this stage by means of cognitive functions such as working memory (Rönnberg et al., 2013). Lastly, sentence-level interpretation takes place at the comprehension stage, which involves further linguistic and cognitive processing (Wingfield and Tun, 2007; Gordon-Salant et al., 2020). With this complex interplay between acoustic and linguistic factors, speech perception can be considered a complex dynamic system: it continuously adapts as it unfolds, and its outcome – a listener’s understanding of a speaker’s intended message – is difficult to predict (Larsen-Freeman, 1997).

### Processing effort in speech perception

In order to better understand the complex dynamic system of speech perception and be able to make predictions about outcomes, it is critical to examine how acoustic, cognitive, and linguistic complexities interact across the multiple stages of speech perception. Prior research indicates that performance on higher-level cognitive and linguistic tasks (e.g., memory encoding, language comprehension) is negatively impacted by acoustic challenges such as poor audibility, background noise, and time compression (e.g., Rabbitt, 1968; Murphy et al., 2006; Wingfield et al., 2006; DeCaro et al., 2016). For example, Wingfield and colleagues (Wingfield et al., 2006) examined listeners’ comprehension of time-compressed speech and found the negative impact of time compression on comprehension was stronger for sentences with more complex syntactic structures. These findings can be interpreted as a downstream effect of acoustic complexity on higher-level processing, as described by the “effortfulness hypothesis” (e.g., Rabbitt, 1991; Pichora-Fuller, 2003; DeCaro et al., 2016). According to this hypothesis, when more mental resources are allocated to enable the auditory system to cope with the acoustic complexity, there are fewer resources available for higher-level cognitive and linguistic tasks. The effortfulness hypothesis provides theoretical support for a domain-general pool of mental resources that can be impacted by both acoustic and cognitive/linguistic complexities and lead to increased processing effort during speech perception under adverse conditions.

In the literature, this processing effort is defined as resources that are purposefully distributed to address problems or challenges while carrying out a task (Pichora-Fuller et al., 2016). Listening effort refers specifically to the processing effort expended while attending to and interpreting information delivered auditorily (McGarrigle et al., 2014). Strauss and Francis (2017) posit a model of processing effort that includes both external (i.e., sensory) and internal (i.e., cognitive) dimensions and argue that the level of effort expended is modulated by external and internal attentional demands. This model can be related to the stages of the speech perception process, with the external dimension primarily located within the perceptual processes of detection and stream segregation stages. The internal dimension, on the other hand, relates to the linguistic and cognitive processing that takes place during the recognition and comprehension stages. Factors related to both the external dimension (e.g., presence of background noise) and the internal dimension (e.g., listener’s working memory capacity) can serve to modulate how much processing effort a listener expends.

Importantly, Strauss and Francis (2017) note that task performance is not solely dependent on the level of effort expended by a listener. Evidence from the literature supports this underlying assumption of their model of processing effort. For example, Norman and Bobrow (1975) described data-based tasks in which allocating additional mental effort is either ineffective or impossible because performance is determined only by the quality of the data available. More recent empirical research has provided converging evidence demonstrating the dissociation between objective measures of effort and behavioral data. In a dual-task paradigm, Gosselin and Gagné (2011) found that older adults performed a sentence recognition task with comparable accuracy to younger adults, but expended greater effort as revealed by poorer performance on a secondary tactile pulse pattern-recognition task. Winn and Teece (2021) found that differences in effort were not reflected by intelligibility accuracy scores. Koelewijn et al. (2018) demonstrated that changes in listener motivation based on the size of a monetary reward for higher accuracy can impact the level of effort expended without affecting performance as measured by intelligibility scores. These data illustrate the importance of measuring processing effort during speech perception under adverse conditions in order to provide additional information about this complex dynamic process.

### Effects of acoustic challenge and processing depth on pupil response

One approach to measuring processing effort is examining task-evoked pupil response. Changes in pupil dilation occur as a central nervous system response during cognitive processing (Beatty, 1982). The measure of pupil dilation can be used as a reflection of changes in processing effort expended while completing a task (Burlingham et al., 2022). While this approach has been used extensively in cognitive psychology research to examine cognitive processing (see van der Wel and van Steenbergen, 2018 for a review), only over the past decade has it gained traction in hearing research as a means of measuring and understanding processing effort during speech perception (see Winn et al., 2018; Zekveld et al., 2018 for reviews). Recent studies using the paradigm of measuring pupil dilation during speech perception tasks have provided substantial evidence for the effects of a variety of perceptual and cognitive factors on task-evoked pupil response. For example, peak pupil dilation was significantly higher with speech recognition in noise than in quiet, which indicates a more effortful process of speech perception with noise (Zekveld and Kramer, 2014). Pupil response is more elevated with masking from a competing talker, and pupils tend to reach a maximum dilation with an SNR (Signal-Noise-Ratio) that is a medium level of difficulty (Wendt et al., 2018).

Although the pupil response observed in prior studies involving speech perception tasks has typically been interpreted as reflecting the acoustic challenge of noise, it is worth noting the pupil response observed during speech perception tasks is not evident in other tasks that do not require cognitive or linguistic processing of the speech content. As an example, no effect of acoustic degradation was found on pupil response in a task requiring recognition of single letters (McCloy et al., 2017). Based on these results, the processing effort evidenced in participants’ pupil responses during speech perception in noise appears to stem from resolving acoustic challenges during higher-level processing of speech content (Strauss and Francis, 2017). This rationale also aligns with evidence that changes in processing depth associated with different speech perception tasks have a significant impact on pupil response. For instance, Kramer and colleagues found that noise-in-noise detection induced lower pupil dilation than word-in-noise identification (Kramer et al., 2013). Similarly, Chapman and Hallowell (2021) found that changes in pupil dilation were only evident when listeners answered comprehension questions about sentences they heard, not when asked to merely listen to the speech without completing any task.

This evidence that listeners expend greater effort during higher-level speech perception tasks in noise as compared to other acoustic tasks in noise converges with a broader body of literature which shows the effect of processing depth on effort. The term “depth of processing” refers to the concept that there are multiple levels at which information can be analyzed (Craik and Lockhart, 1972). An example of two tasks requiring different depths of processing based on the same visually presented sentence is a reading task, which requires linguistic processing, versus a letter detection task, which only requires visual analysis (e.g., Meng et al., 2020). Tasks that require deeper cognitive and linguistic processing induce more effort as measured by a variety of measures including pupil response, reaction time, and performance on a secondary task (e.g., Hyönä et al., 1995; Picou and Ricketts, 2014; Wu et al., 2014). It is worth noting that this relationship between processing depth and effort only holds when the listener is engaged in the task. Wu et al. (2016) did not find an effect of task difficulty with a dual-task measure of effort, which could reflect participant disengagement from the primary task. Pupil response measures by Zekveld et al. (2014) revealed diminished effort when the task was too difficult for the listener to engage.

Although these data demonstrate how the acoustic challenge of noise and the depth of linguistic processing of speech can affect pupil response separately, little is known about the relative contributions of and interactions between these factors when speech processing takes place under adverse conditions. One study that measured reaction time in a dual-task paradigm to measure processing effort revealed an interaction between processing depth and background noise in a group of younger adults (Picou and Ricketts, 2014). The effect of noise on reaction time was stronger for the task that required linguistic processing as compared to tasks that required responses based on presentation of simple visual stimuli. As dual-task paradigms used across different studies may have intrinsic variability and be difficult to compare due to differences in the secondary task (e.g., Wu et al., 2016; Schoof and Souza, 2018), further evidence is needed to demonstrate the interaction between noise and processing depth utilizing a non-dual task paradigm and an objective measure of processing effort such as pupil response.

### Effects of dynamic pitch alteration in noise on pupil response

While much is known about the effects of noise on speech perception, the field of hearing science has recently begun to recognize the effects of other acoustic variables on speech perception (Keidser et al., 2020). Dynamic pitch, or pitch variations in speech, has been shown to be an important cue for speech perception in noise (Binns and Culling, 2007). In real-life communication, flattened and altered dynamic pitch cues have often been observed in dysarthric speech (e.g., Schlenck et al., 1993). While this acoustic alteration has significant impact on speech recognition, particularly in noise (e.g., Laures and Weismer, 1999; Bunton et al., 2001; Miller et al., 2010; Calandruccio et al., 2019; Shen, 2021a), no evidence was available regarding its impact on processing effort. A small preliminary dataset suggested that dynamic pitch alteration induced heightened pupil response (Shen, 2021b) but more data were needed to understand the nature of this phenomenon. Following the rationale that pupil response could reflect both linguistic processing effort and the perceptual effort due to acoustic challenges (Zekveld et al., 2018), it is unclear whether these two factors were both at play when stronger pupil response was observed with altered dynamic pitch in a speech recognition in noise task.

One explanation based on perceptual mechanisms for the results of Shen (2021b) is that dynamic pitch alteration created an acoustic signal that was unfamiliar to the listeners and the observed pupil response reflected the perceptual novelty effect (e.g., Liao et al., 2016; Beukema et al., 2019). For example, in a study that used auditory oddball paradigm, Liao et al. (2016) found white noise bursts and tones with different pitches in a tone sequence elicited pupil dilation. Another possibility is that intonation patterns in speech are particularly important for speech recognition in noise (Cutler, 1976; Nooteboom et al., 1978). When pitch alteration changes the natural intonation pattern, linguistic processing in noise becomes more difficult. Notably, this hypothesized effect of altered intonation only applies to speech processing in noise; intonation cues are not critical to understanding speech in quiet (Wingfield et al., 1989).

### Study objectives

The primary objective of this study was to examine the interactions of the acoustic challenge of noise and processing depth required by a task and the resulting effects on processing effort, reflected by pupil response, in a group of younger adults with typical hearing. A tiredness judgment task was developed as a comparison task to speech recognition. For this task, participants make a judgment about whether the speaker sounds tired or not tired based on their perception of the speech sound without processing of the linguistic content of speech. In terms of processing depth, this tiredness judgment task involves a shallower level of processing than the speech recognition task because it only requires listening to speech with background noise without linguistic processing of the speech content. With respect to the acoustic challenge, non-speech noise was compared with a quiet condition for both the tiredness judgment and the speech recognition tasks. Based on prior research examining the effects of noise and processing depth on processing effort, pupil response (as measured by peak pupil dilation) was anticipated to be stronger in noise than in quiet, particularly during speech recognition than the tiredness judgment task. The main research question focused on understanding how depth of processing modulates the impact of background noise on processing effort. In other words, can peak pupil dilation data shed light on the relative effects of the processing depth (i.e., processing of the meaning of a spoken message) and the acoustic challenge (i.e., perceiving speech in noise)? To examine these questions, two experiments were designed involving speech perception tasks (a higher-level processing speech recognition task and a lower-level processing tiredness judgment task) in noise in Experiment 1 and without noise in Experiment 2.

The secondary objective of this study was to determine whether the effect of pitch alteration on pupil dilation observed in the preliminary data of Shen (2021b) was due to effortful linguistic processing of speech with altered intonation in noise or the perceptual novelty effect due to pitch contours perceived as unnatural. The tiredness judgment task included in Experiments 1 and 2 was designed to limit the need for deep linguistic processing as listeners were merely asked to make a subjective judgment about whether the speaker sounded tired in each trial. It was first hypothesized that if the pupil dilation observed was due to the perceptual novelty effect of pitch alteration, peak pupil dilation would be higher when participants listened to pitch-altered speech (e.g., Liao et al., 2016) during the tiredness judgment task in comparison with non-altered speech. To further test this hypothesis, Experiment 2 included both the speech recognition and the tiredness judgment tasks without background noise, and two primary research questions were explored. The first question was, does pitch effect persist in a task that does not require processing of the speech content? The second question was, does pitch effect persist in quiet when speech perception is not effortful? The answer to these questions can illuminate whether the mechanism is related to perception of acoustic signal or processing of the linguistic content in noise. If the effect of pitch alteration on pupil response stems from perceptual novelty, this effect should impact the early processing stage of acoustic detection (Liao et al., 2016). As a result, the effect of pitch alteration on pupil response would persist across both speech recognition and tiredness judgment tasks in quiet. Alternatively, if the effect of pitch alteration were due to altered intonation that hindered linguistic processing in noise, this effect would not be observed when speech recognition occurs in quiet. To summarize, if the effect of pitch alteration on pupil response is due to perceptual novelty, the effect would be expected in both types of tasks, as well as in both noise and quiet. If the effect of pitch alteration on pupil response is due to difficulties with processing speech with atypical prosody in noise, then the effect is only expected in speech recognition in noise.

The knowledge gained from the present study has theoretical implications in that it can improve understanding of the interactions among the multiple stages of the speech perception process when it takes place under adverse conditions (Gordon-Salant et al., 2020), with the goal of effectively capturing and predicting individuals’ response to a wide range of environmental and listener variables (Pichora-Fuller et al., 2016). As the field of audiology strives to improve ecological validity of clinical diagnostic and intervention protocols (Keidser et al., 2020), the present study aims to move the field toward this goal. This work has further clinical implications in that it can inform the development of clinical tests and interventions with populations that experience speech perception challenges due to multiple interacting factors (e.g., older adults with hearing loss and cognitive decline).

## Materials and methods

### Participants

Twenty-one young participants were included in Experiment 1 (mean age 19.85 years with range 18–22 years). Eighteen participants self-identified as female and three as male. Twenty-three young participants were included in Experiment 2 (mean age 20.43 years with range 18–31 years). Seventeen participants self-identified as female and six as male. One participant’s data were removed from analysis due to technical errors. All participants had normal hearing as measured by audiometric testing (with Pure Tone Average across 0.5, 1, 2 k Hz < 20 dB HL). All participants were native speakers of General American English recruited from the Temple University community and were either paid or awarded course credit for their time. The study protocol was approved by the Institutional Review Board of Temple University.

### Stimuli

The same stimuli set from Shen (2021b) was used in this study. The sentences were originally taken from IEEE/Harvard sentence corpus (Rothauser et al., 1969), with five keywords in each sentence. The stimuli were produced by a female native speaker of General American English. Following the same alteration strategies used in previous research (Binns and Culling, 2007; Shen and Souza, 2017), the sentences were resynthesized to have altered dynamic pitch cues using the Praat program (Boersma and Weenink, 2013). Three dynamic pitch conditions were created: original dynamic pitch (the original pitch contour, stimuli processed using the same resynthesis method as the strengthened/weakened pitch stimuli), strengthened dynamic pitch (1.4 times original pitch contour), and weakened dynamic pitch (0.5 times original pitch contour). Mean sentence duration was 2,668 msec with a range of 1946 to 3,292 msec. The noise stimulus was non-speech noise that preserved the temporal and spectral characteristics of 2-talker babble (ICRA, 2-talker noise; Dreschler et al., 2001).

### Experiment design

In Experiment 1, the sentences were embedded in noise with a signal-to-noise-ratio of-5 dB SNR, which was used based on the data from Shen (2021b), with speech recognition accuracy in the range of 70–90% correctly recognized keywords. In Experiment 2, the same set of sentences were presented in quiet.

In each of the two experiments, there were 144 trials in total distributed across 6 conditions with a full combination of pitch and task conditions. There were 3 pitch conditions (original, strong, weak) and 2 task conditions (speech recognition and tiredness judgment), with 2 blocks of 12 trials for each condition. The pitch condition was mixed in each block with order randomized across participants.

For half of the testing session in both Experiments (Experiment 1 in noise, Experiment 2 in quiet), participants completed the speech recognition task, in which participants were asked to repeat back the sentence after listening to it. For the other half, participants completed the tiredness judgment task, in which participants were asked to pay attention to the voice of the talker in each sentence and report whether the talker sounded tired. Participants were instructed to provide verbal responses, saying “tired” or “not tired” based on their subjective judgment of the voice. The task was blocked in both experiments with order of the two tasks counterbalanced across participants.

Prior to testing in each block, the participants had a brief practice session consisting of 6 trials (with 2 trials for each pitch condition) to familiarize them with the procedure. Each experiment took approximately 90 min in total, and participants were given a break every 3 blocks to prevent fatigue.

### Pupillometry procedure

The pupil diameter data were collected using an Eyelink 1,000 plus eye-tracker in remote mode with head support. The sampling rate was 1,000 Hz and the left eye was tracked. During testing, the participant was seated in a dimly lit double-walled sound booth in front of a BenQ Zowie LCD monitor, with the distance between eye and screen set at 58 cm. Based on piloting and previous data (Shen, 2021b), the color of the screen was set to be gray to avoid outer limits of the range of pupil diameter. The luminance measure was 37 lux at eye position.

The experiment was implemented with a customized program using Eyelink Toolbox (Cornelissen et al., 2002) in MATLAB. Auditory stimuli were presented over a Sennheiser HD-25 headphone at 65 dBA. For each trial, a red “X” sign was first presented at the center of the screen. The participant was instructed to look at the sign once it appeared. After 2,000 ms of silence (baseline period), an audio stimulus was played. Because the sentences varied in their duration, they were offset aligned to facilitate the comparison of the peak pupil dilation, which has been demonstrated by previous studies to occur near the sentence offset (e.g., Winn et al., 2015; Shen, 2021b). Each trial began with background noise (Experiment 1), or quiet (Experiment 2) before the sentence started. The sentence began about 2000 ms after the noise or quiet onset, depending on the sentence length. The total length of each audio stimulus was 4,500 ms. There was a retention period of 2000 ms after the stimulus finished playing. After the retention period, the red “X” sign was replaced by a green “X” sign that was of equal luminance, and the participant was instructed to provide a verbal response only after the green “X” sign appeared. Once the participant completed a response, the tester terminated the trial with a key press, and a gray box appeared at the center of the screen after 1,000 ms of silence. The participant was instructed to rest their eyes when the gray box was on the screen. The resting period lasted for 5,000 ms before the next trial started. Pupil dilation data were recorded continuously throughout the entire session. The data file was tagged with time stamps that were synchronized with each of these visual and auditory events.

Although the subject matter of the spoken material was largely neutral after removal of items with high emotional valence (Shen, 2021b), offline semantic ratings of each sentence were collected to control for emotional variability across sentences after pupillometry testing. Participants were instructed to provide ratings (positive, negative, or neutral) based on the content of each sentence.

### Data processing and analysis

Pupil diameter data were pre-processed using R (Version 3.2.1) with GazeR library (Geller et al., 2020). As pupil diameter is shown to be altered by fixation location (Gagl et al., 2011; Hayes and Petrov, 2016), a center area of the screen was defined by ±8° horizontal and ± 6° vertical to obtain pupil alteration rate less than 5% (Hayes and Petrov, 2016). Pupil diameter data were removed from further analysis when the fixation location was outside of this area, resulting in removal of 4% of data points. Using the Eyelink blink detection algorithm, missing pupil diameter data were marked as blinking. De-blinking was implemented between 100 ms before the blink and 100 ms after the blink. The curve was linearly interpolated and smoothed using a 20-point moving average. Three participants’ data were removed from further analysis due to excessive blinking (more than 20% of trials have more than 15% of missing data points). Following a method that has been suggested in the pupillometry literature (e.g., Winn et al., 2018; Geller et al., 2020), the pupil diameter data were downsampled to 10 Hz (by aggregating data every 100 ms) before statistical analysis.

The dependent measure was peak pupil dilation relative to individuals’ baseline pupil diameter of each trial (by subtraction). Baseline pupil diameter was calculated based on mean pupil diameter recorded during the 1,000 ms silent period before the sound onset. The window of –1,000 to 1,500 ms relative to sentence offset was used for calculating peak pupil dilation. This window was chosen based on data from previous studies using the same stimuli set (e.g., Winn et al., 2015; Shen, 2021b) and is thought to reflect the cumulative processing effort expended approaching and following the end of sentence. As the sentences were offset aligned and varied slightly in length, there was a segment of either noise or quiet (depending on the noise condition) lasting approximately 1,000–1,500 ms before each sentence onset. To normalize the peak pupil dilation across the noise vs. quiet conditions and control for the effect of noise before sentence onset, mean pupil dilation in the window before sentence onset (−4,000 to –3,500 ms relative to sentence offset) was calculated for each condition (task/noise/pitch) and subtracted from peak pupil dilation in the analyses involving both noise and quiet conditions.

## Results

**Figure 1:**
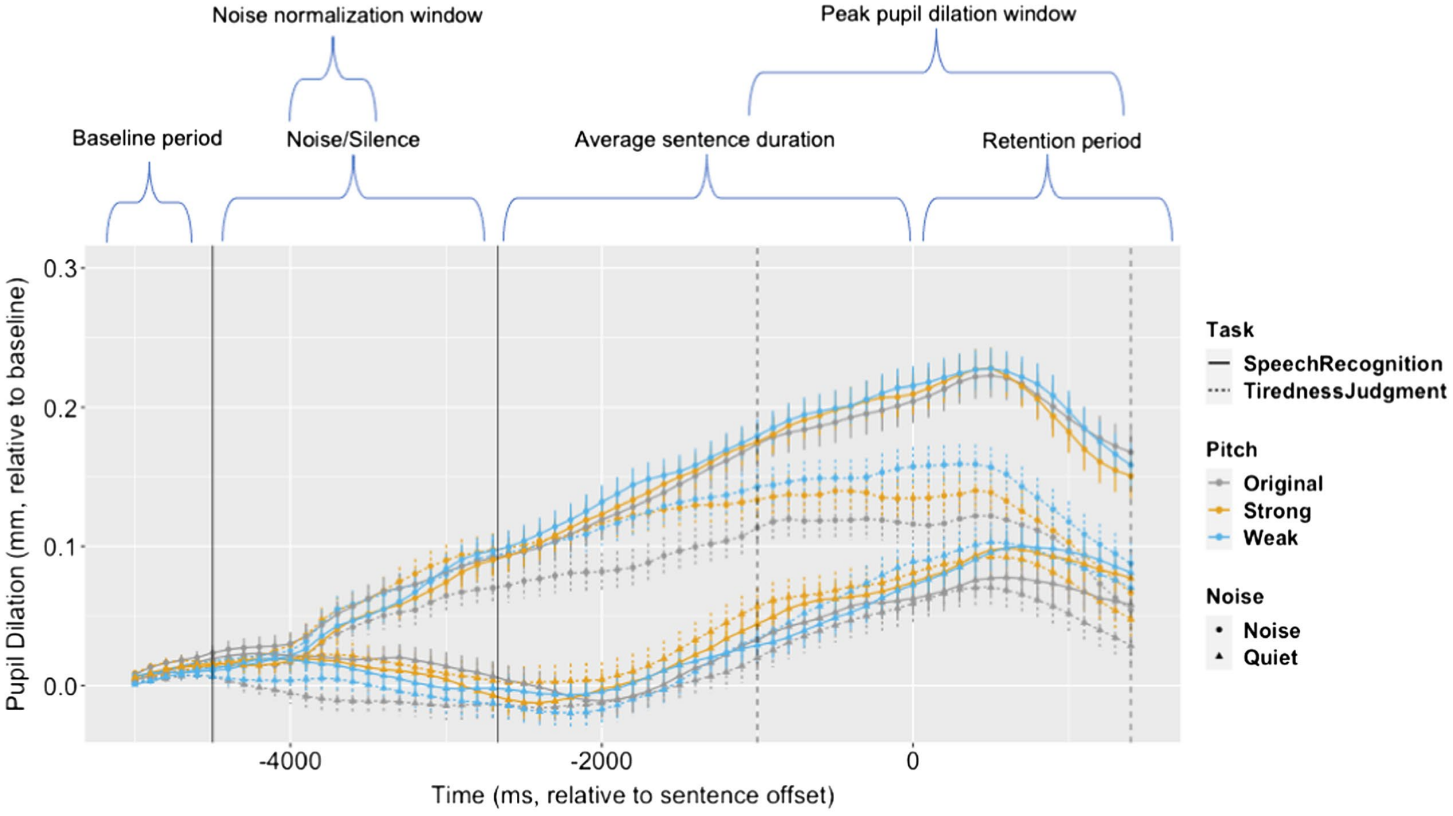
Pupil dilation relative to baseline during the time window of -5,000ms to +1,500ms relative to sentence offset. Error bars: ± standard error. The vertical dotted lines mark the onset and offset of the time window for peak pupil dilation measure. The vertical solid lines mark the onset of sound stimuli and the average onset of sentences. The labels above the graph indicate different events and analysis windows during the trial.

Linear mixed effect models (Baayen, 2008) were used to identify the effect of noise in the speech recognition task vs. the tiredness judgment task on the dependent variable of peak pupil dilation relative to baseline level for each trial. Figure 1 presents pupil dilation (i.e., the pupil diameter relative to baseline) of all conditions, during the time window of –5,000 ms to +1,500 ms relative to sentence offset, collapsed across participants.

A model that included all three factors of task (speech recognition vs. tiredness judgment), noise (quiet vs. noise), and pitch (original vs. strong vs. weak) was first constructed to test the main effect of noise and its interaction with task. Following the recommended practice of using linear mixed effect models (Meteyard and Davies, 2020), the model was built based on our study objectives, with fixed effects for all three variables (task, noise, and pitch) added first, followed by interaction among these variables. In all models, we included variables for trial order and semantic rating and allowed for by-participant random intercept and slope and by-item random intercept and slope (Barr et al., 2013). Model comparison results are reported in Table 1. The fixed factors of noise and task and their interaction were found to significantly improve model fit. In the final model, the peak pupil dilation was lower in tiredness judgment compared to speech recognition (*β* = 75.70, *p* = 0.01). The effect of task was significantly modulated by the presence of background noise (*β* = −186.6, *p* < 0.01), with larger difference in peak pupil dilation between the two tasks in noise than in quiet (Table 2).

**Table 1:** Model comparison and the model building process for the effect noise and task on peak pupil dilation.

Sampling Units		N total observations = 135,573 N Subjects = 40 N items = 144									
Model specification	Model name	Nested/simpler model	Fixed effects added	Random effects		Model fit				LFT test against nested	
				Participants	Items	AIC	BIC	LL	*df*	*df*	*X* ^2^
RE only		–	–								
RE only		–	–	Intercept + Slope	Intercept + Slope	2,101,986	2,102,094	−1,050,982	10		
FE main effects	Main effects 1	–	Trial Order + Semantic Rating + Task + Noise + Pitch	Intercept + Slope	Intercept + Slope	2,101,912	2,102,059	−1,050,941	14	4	81.387
FE two-way interactions	Model 2	Model 1	Trial Order + Semantic Rating + (Task × Noise) + Pitch	Intercept + Slope	Intercept + Slope	2,101,904	2,102,062	−1,050,936	15	1	9.6597
FE two-way interactions	Model 3	Model 1	Trial Order + Semantic Rating + Task + (Noise × Pitch)	Intercept + Slope	Intercept + Slope	2,101,912	2,102,079	−1,050,939	16	1	1.00
FE three-way interaction	Model 4	Model 2/3	Trial Order + Semantic Rating + (Task × Noise × Pitch)	Intercept + Slope	Intercept + Slope	2,101,896	2,102,085	−1,050,913	21	5	53.0571

**Table 2:** Final model for the effect of noise and task on peak pupil dilation.

	Beta	SE	*t*-value	*p*
Intercept	269.9	40.01	6.744	<0.001
Trial order	−6.540	0.225	−29.007	<0.001
Semantic Rating 1	−23.89	5.110	−4.676	<0.001
Semantic Rating 2	−6.784	4.761	−1.425	0.154
Task	75.70	28.22	2.682	0.010
Noise	−145.3	79.07	−1.837	0.073
Pitch 1	11.24	3.850	2.921	<0.01
Pitch 2	31.39	3.837	8.181	<0.001
Task × Noise	−186.6	56.47	−3.305	<0.01
Noise × Pitch 1	−8.474	7.700	−1.100	>0.1
Noise × Pitch 2	6.683	7.671	0.871	>0.1
Task × Pitch 1	44.19	7.686	5.749	<0.001
Task × Pitch 2	6.452	7.694	0.838	>0.1
Task × Noise × Pitch 1	7.160	15.36	0.466	0.641
Task × Noise × Pitch 2	−18.59	15.39	−1.208	0.226
***Random effects***				
	**Variance**	***S.D.***	**Correlation**	
Participant (Intercept)	59,922	244.8		
Participant task (Slope)	31,090	176.3	−0.25	
Items (Intercept)	11,139	105.5		
Item noise (Slope)	27,246	15.1	−0.16	

Key: *p*-values for fixed effects calculated using Satterthwaites approximations. Final Model equation: PupilSize ~ (1 + Task| Subject) + (1 + Noise|Item) + TrialOrder + Semantic Rating + (Task × Noise × Pitch).

In the second part of the analysis, we built two specific models and tested the effects of pitch alteration on peak pupil dilation data in tiredness judgment task and in quiet, respectively. Similarly to the first analysis, we used mixed-effects linear regression models with the dependent variable of peak pupil dilation. The original dynamic pitch condition was treated as baseline and parameters were estimated for the strengthened and weakened dynamic pitch conditions. The model for the tiredness judgment task included fixed effects for pitch and noise condition. Semantic rating and trial order as fixed factors, by-participant random intercept, by-item random intercept and slope were also included in the model. Model comparison results are reported in Supplement Table 1. We saw significant increases in peak pupil dilation in pitch-altered conditions as compared to original pitch (stronger pitch: *β* = 28.18, *p* < 0.001; weaker pitch: *β* = 57.15, *p* < 0.001). No significant effect of noise was found on the tiredness judgment performance (*β* = −132.40, *p* = 0.15), suggesting the processing effort in the tiredness judgment task is not affected by background noise. The interactions between pitch and noise were significant for both stronger and weaker pitch (see Table 3). The increased pupil dilation differences between original and stronger/weaker pitch conditions in quiet indicate the increased perceptual arousal without noise.

**Table 3:** Final model for the analysis of dynamic pitch/task on peak pupil dilation (Tiredness Judgement Task).

	Beta	SE	*t*-value	*p*
Intercept	268.785	46.023	5.840	<0.001
Trial order	−5.2939	0.3328	−15.906	<0.001
Semantic Rating 1	−51.1662	7.319	−6.990	<0.001
Semantic Rating 2	−50.3475	6.853	−7.346	<0.001
Noise	−132.408	90.515	−1.463	0.150
Pitch 1	28.186	5.475	5.147	<0.001
Pitch 2	57.153	5.458	10.471	<0.001
Noise × Pitch 1	30.278	10.938	2.768	<0.01
Noise × Pitch 2	21.915	10.912	2.008	<0.05
***Random effects***				
	**Variance**	***S.D.***	**Correlation**	
Participant (Intercept)	77,109	277.7		
Items (Intercept)	21,223	145.7		
Items noise (Slope)	54,748	234.0	0.04	

Key: *p*-values for fixed effects calculated using Satterthwaites approximations. Final Model equation: PupilSize ~ (1|Subject) + (1 + Noise|Item) + TrialOrder + Semantic Rating + (Noise × Pitch).

The model for the quiet condition alone included fixed effects for pitch and task. Semantic rating and trial order as fixed factors, by-participant random intercept and slope, by-item random intercept were also included in the model. Model comparison results are reported in Supplement Table 2. There were significant pitch effects, with pupil dilation increasing when participants were exposed to weaker dynamic pitch (*β* = 46.158, *p* < 0.001) and stronger dynamic pitch (*β* = 40.003, *p* = 0.001) as compared to baseline original pitch. The effect of task was also significant (*β* = 5.367, *p* < 0.001), indicating higher effort in speech recognition task than in tiredness judgment task. There was also evidence that both pitch conditions interact with task, with increased pupil dilation differences between original and stronger/weaker pitch conditions in tiredness judgment task (see Table 4).

**Table 4:** Final model for the analysis of dynamic pitch/task on peak pupil dilation (Quiet only).

	Beta	SE	*t*-value	*p*
Intercept	192.58	47.746	24.49	<0.001
Trial order	−4.484	0.305	−14.69	<0.001
Semantic Rating 1	−24.60	6.909	−3.561	<0.001
Semantic Rating 2	1.788	6.509	0.275	0.784
Task	5.367	33.189	−14.689	<0.001
Pitch 1	40.003	5.233	7.644	<0.001
Pitch 2	46.158	5.205	8.867	<0.001
Task × Pitch 1	−22.422	10.482	−2.139	<0.05
Task × Pitch 2	−37.359	10.441	−3.578	<0.001
***Random effects***				
	**Variance**	***S.D.***	**Correlation**	
Participant (Intercept)	47,402	217.7		
Participant task (Slope)	23,782	154.2	−0.40	
Items (Intercept)	15,180	123.2		

Key: *p*-values for fixed effects calculated using Satterthwaites approximations. Model equation: PupilSize ~ (1 + Task|Subject) + (1|Item) + TrialOrder + Semantic Rating + (Task × Pitch).

**Figure 2:**
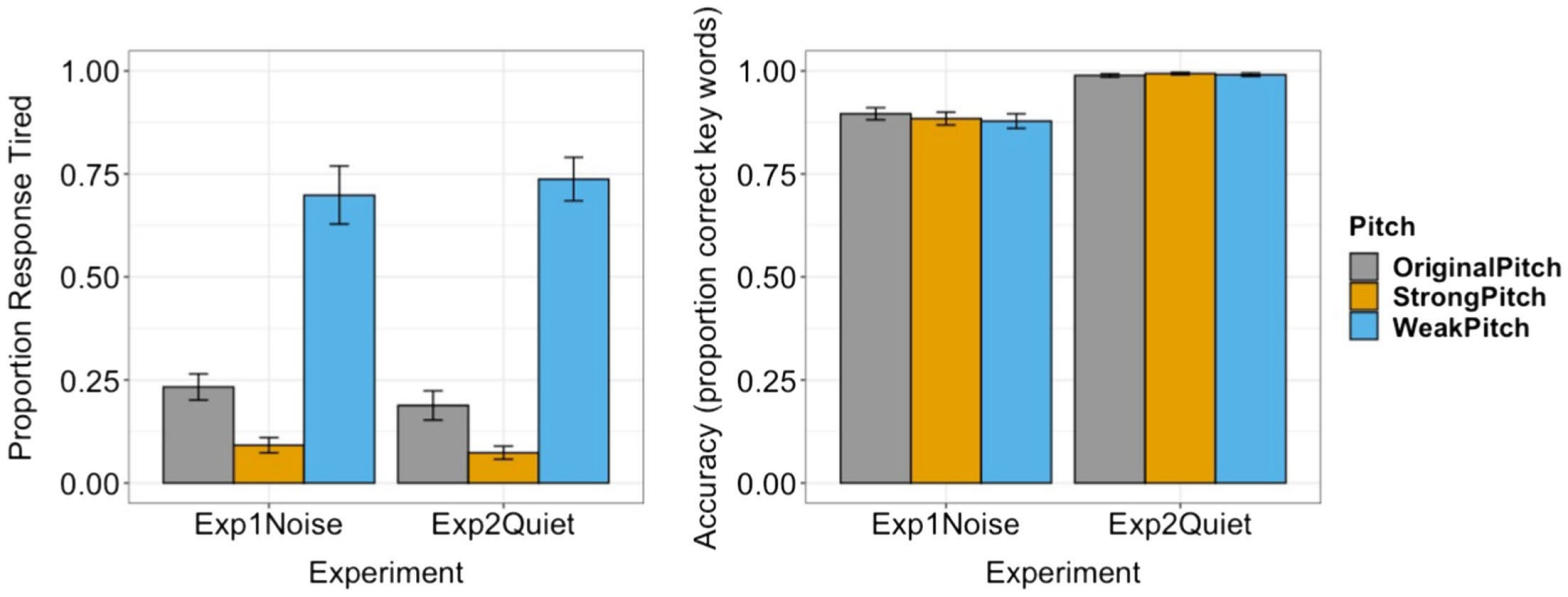
Behavioral data of tiredness judgment (left panel) and speech recognition (right panel). Error bars: ± standard error.

In addition, group data on behavioral performance for all conditions are reported in Figure 2.

## Discussion

### Interplay between acoustic challenge and processing depth

With data from two experiments, the present study demonstrated the complex interaction between acoustic challenges and linguistic processing during speech perception under adverse conditions. As expected, stronger pupil dilation was observed when participants carried out the speech recognition task with background noise present, indicating additional effort expended to cope with the acoustic challenge of noise. Critically, there was an effect of noise during the speech recognition task but not the tiredness perception task, as indicated by a significant interaction between task and noise conditions and a non-significant main effect of noise. These data are consistent with previous findings (Picou and Ricketts, 2014; Chapman and Hallowell, 2021) and demonstrate that the processing effort due to background noise is modulated by the depth of processing required by the task, even when both tasks require the same level of effort in quiet. When the task requires greater higher-level processing of linguistic content, it requires more effort to complete with background noise. This effect of noise, however, was not observed with the tiredness judgment task, which requires shallower processing given that the judgment can be made based solely on the speech sounds. This finding aligns with results from Kramer et al. (2013) showing that a word recognition in noise task took more cognitive effort, as measured by pupil response, than a perception task involving detecting a noise burst in noise.

From a methodological perspective, it is important to note that the present study used the same type of speech stimuli in both tasks to rule out the confounding factor of differences in auditory processing between different types of stimuli (e.g., tones vs. words). Therefore, the observed difference in processing effort between the speech recognition and tiredness judgment tasks in noise can be attributed to the linguistic processing required to recognize speech content in noise. As a result, these data indicate a non-additive relationship between the effort due to acoustic challenge (i.e., noise) and processing depth, as demonstrated by task-evoked pupil response. These results are also consistent with those from Meng et al. (2020), which demonstrated a similar effect of processing depth on the impact of background noise during reading tasks. These converging data extend the effortfulness hypothesis (Rabbitt, 1991; DeCaro et al., 2016) and demonstrate that the processing depth of the target speech can modulate the effect of background noise on processing effort. Compared to previous work, the present study contributes to the literature by using two tasks that differ in processing depth, but both involve functional speech communication (i.e., one pertains to recognition of the linguistic content, the other to the perception of the paralinguistic aspect of speaker’s level of tiredness).

### The nature of dynamic pitch alteration effect

Building upon the findings of Shen (2021b), the secondary goal of this study was to determine whether the effect of pitch alteration on peak pupil dilation was due to more effortful linguistic processing because of the altered intonation or a perceptual novelty effect in response to unnatural pitch contours. This question was originally motivated by the altered dynamic pitch cues in dysarthria speech (Schlenck et al., 1993; Bunton et al., 2001) and has clinical impact with respect to understanding how this type of acoustic alteration is perceived by listeners with and without background noise. When considered together, data from the two experiments support a perceptual novelty effect hypothesis. First, the pitch alteration effect was evident in the tiredness judgment task, in which processing of linguistic content was not necessary. Based on the multistage model of speech perception in noise (Gordon-Salant et al., 2020), the perceptual novelty effect should occur in an early stage of speech perception when the acoustic signal is perceived (e.g., Liao et al., 2016). Given that this process happens regardless of task type, the perceptual novelty effect should impact pupil response across the different tasks of speech recognition and tiredness judgment. Furthermore, when the speech recognition task was completed in quiet, the effort associated with processing of the linguistic content in background noise was eliminated. Although intonation can facilitate linguistic processing under adverse conditions, it is not necessary for speech understanding in quiet (Wingfield et al., 1989). Therefore, the altered intonation should not cause an elevation in effort for linguistic processing in quiet. The elevated pupil dilation associated with altered dynamic pitch, however, was still observed in the speech recognition task in quiet. A plausible explanation of these results is a perceptual novelty account in the early stages of the speech perception process, meaning the altered pitch cues affected perception of the speech sound.

Interestingly, the data from the present study showed a slightly different pattern of pitch alteration effects as compared to the results of Shen (2021b) described above. Pupil response had a positive relationship with dynamic pitch strength in the Shen (2021b) results, with the lowest pupil response observed in the weakened pitch condition and the highest pupil response in the strengthened pitch condition. The present data, however, demonstrated heightened pupil response to both strengthened and weakened pitch, which may further support a perceptual novelty explanation (e.g., Liao et al., 2016; Beukema et al., 2019). In the present study, the altered dynamic pitch in speech was unfamiliar (and likely unnatural) to the listeners and could have induced a novelty effect regardless of task.

Across the datasets between the present study and Shen (2021b), the differences in weakened pitch condition can potentially be explained by the methodological differences of mixed versus blocked design (e.g., Koelewijn et al., 2015). Sentences with different pitch alterations (i.e., strengthened, weakened, original) were presented in separate blocks in Shen (2021b) but were mixed in the present study; therefore, the reduced pupil response to weakened dynamic pitch cues in the Shen (2021b) data was likely due to stronger perceptual adaptation to weakened dynamic pitch pattern (e.g., Anstis and Saida, 1985; Hubbard and Assmann, 2013). When the pitch conditions were mixed in the current paradigm, listeners did not adapt to weakened dynamic pitch and therefore had increased pupil responses to each type of pitch alteration, which changed in every trial and induced greater novelty effects. There is also an alternative explanation for the decreased pupil dilation for weakened pitch in a block design, which is not mutually exclusive to the previous one. It is possible that when participants anticipated the unfamiliar stimuli with weakened dynamic pitch in a blocked design, the anticipation of an upcoming challenge may have resulted in an elevated pupil baseline. As peak pupil dilation measure is derived from pupil diameter measure corrected for baseline, a heightened baseline could potentially lead to a reduced pupil dilation measure (e.g., Winn et al., 2018). While it was not within the scope of this present study to test these possibilities, they should be examined in future research.

### Implications and future directions

In addition to their theoretical contributions, the current findings also highlight a few issues that bear clinical significance. First, while data were only collected from younger adult participants with typical hearing in the current study, an important question for further research is how the listener factors of age-related hearing loss and cognitive aging may influence effort, as indicated by pupil response, during speech perception under adverse conditions. As an example, speech understanding in background noise is one of the most challenging issues for older adults with hearing loss (Humes et al., 2020). While the research in this area has been largely focused on the impact of the acoustic challenge of noise on speech perception, there is limited evidence showing how this difficulty is related to and can be modulated by the processes of linguistic and cognitive processing (Gordon-Salant et al., 2020). We know real-life communication can become even more challenging due to both acoustic and linguistic factors. For example, if a listener were trying to comprehend a discussion of an unfamiliar and complex topic taking place in a noisy environment, the listener cannot rely on prior knowledge to cope with the background noise; perceptual and cognitive processes must take place simultaneously to cope with the coinciding perceptual and cognitive difficulties. Greater understanding of the combined effects of acoustic and cognitive challenges on speech perception is critical for devising new clinical diagnostic protocols with improved ecological validity to represent a range of real-life challenges. The present study contributes to this work by taking an initial step toward demonstrating the interaction between the acoustic challenge of noise and processing depth when these factors affect processing effort. Future research should employ tasks that better resemble real-life communication and by examining the impact of listener factors (e.g., hearing loss, cognitive aging) in complex communicative scenarios.

Furthermore, while it is already known that speech recognition in noise is more difficult with altered dynamic pitch in dysarthric speech (Laures and Weismer, 1999; Bunton et al., 2001), no data previously existed regarding the mechanism behind the impact of this type of acoustic alteration on speech perception. Our results provide new evidence and suggest the altered dynamic pitch can induce a perceptual novelty effect that may affect perception of speech sound across different tasks and regardless of noise. As the pitch alteration in the present study was implemented by a generic algorithm instead of what is measured in dysarthric speech, the current finding should be examined in future research using speech stimuli with more realistic pitch alteration.

One limitation of this study is the variability in performance across participants with respect to intelligibility accuracy. While this variability in behavioral data is largely due to the use of a fixed SNR for all participants, this methodology choice was based on the data from the previous study (Shen, 2021b). Although variability in the behavioral data of speech recognition accuracy was comparable across the previous data set and the present one, this behavioral measure was not found to be a significant predictor for peak pupil dilation data (Shen, 2021b). Additionally, using a fixed SNR rather than an adaptive SNR may be a more ecologically valid approach. In many real-world conversational contexts, the background noise in the environment cannot be adjusted based on the needs of a listener. Because the present study used a fixed SNR that is based on preliminary data for inducing a specific range of recognition performance (70–90% accuracy), this SNR is more adverse than those frequently observed in real-life scenarios (Smeds et al., 2015). For these reasons, future work should consider using SNR that is closer to that is observed in real-life scenarios and customized for each individual to equalize behavioral performance across participants.

Another methodological issue to note is the normalization of pupil dilation across noise and quiet conditions. In the present study, the sentences were offset aligned and therefore had a short segment of noise or silence before sentence onset. This method was used to ensure the same trial duration regardless of sentence length, because trial duration is a factor that may increase pupil dilation (e.g., Burlingham et al., 2022). On the other hand, this offset alignment method inevitably leads to a pre-stimulus acoustic difference, which can result in a difference in pupil dilation at sentence onset between the noise and quiet conditions. We have adopted a solution of normalizing the pupil dilation data using a condition-specific normalization window to estimate the effect of noise vs. silence. It should be noted that this practice of parsing the noise effect out of the final pupil response by subtracting the pupil response after noise onset and before sentence offset (e.g., Mathôt et al., 2018) is currently being debated in the literature. While some research suggests that task-evoked pupil response scales linearly (e.g., Reilly et al., 2019; Denison et al., 2020), Burlingham et al. (2022) argue that pupil dilation is not a linear system that is time-locked to task events, and it may not respond to tasks or stimuli in an additive manner. Future research is needed to determine the most effective approach to teasing apart the effects from noise in isolation versus during speech perception.

In summary, the present study examined how processing effort is influenced by the complex interaction between acoustic challenges and processing depth during speech perception with background noise. Pupil dilation data from younger adults with typical hearing demonstrated that the impact of background noise on processing effort is modulated by depth of processing. Background noise increases effort only when linguistic processing is involved, but not when making a tiredness judgment without the need for linguistic processing. Regarding dynamic pitch alteration, the pupil dilation data support a perceptual novelty hypothesis instead of an effortful linguistic processing one, meaning altered dynamic pitch cues make speech sound unnatural. These findings extend current theories of speech perception under adverse conditions by shedding light on the way in which processing effort is influenced by the interaction between acoustic challenges and linguistic processing. The study also provides a foundation for future work to investigate this complex interaction in clinical populations who experience both hearing and cognitive challenges.

## Data availability statement

The raw data supporting the conclusions of this article will be made available by the authors, without undue reservation.

## Ethics statement

The studies involving human participants were reviewed and approved by Institutional Review Board, Temple University. The patients/participants provided their written informed consent to participate in this study.

## Author contributions

JS and LF contributed to conception and design of the study. JS and EK performed the statistical analysis. JS wrote the first draft of the manuscript. LF and EK wrote sections of the manuscript. All authors contributed to manuscript revision, read, and approved the submitted version.

## Funding

This work was supported by NIH Grant R21DC017560 to JS.

## Conflict of interest

The authors declare that the research was conducted in the absence of any commercial or financial relationships that could be construed as a potential conflict of interest.

## Publisher’s note

All claims expressed in this article are solely those of the authors and do not necessarily represent those of their affiliated organizations, or those of the publisher, the editors and the reviewers. Any product that may be evaluated in this article, or claim that may be made by its manufacturer, is not guaranteed or endorsed by the publisher.
