# Supplementary material for: Interactions between acoustic challenges and processing depth in speech perception as measured by task-evoked pupil response
Source: Front Psychol. 2022 Oct 25;13:959638. doi: 10.3389/fpsyg.2022.959638 (PMC9641013; doi:10.3389/fpsyg.2022.959638)
Supplement: Supplementary file 1 [file Table_1.DOCX]

| **Supplement Table 1. Model comparison and the model building process for the effect of dynamic pitch/noise on peak pupil dilation: Tiredness Judgment Task** | | | | | | | | | | | | |
| --- | --- | --- | --- | --- | --- | --- | --- | --- | --- | --- | --- | --- |
| **Sampling Units** | | **N total observations = 68249**  **N Subjects = 40**  **N items = 144** | | | | | | | | | | |
|  | | | | | | | | | | | | |
| **Model specification** | **Model name** | **Nested/simpler model** | **Fixed Effects Added** |  | **Random Effects** | | **Model Fit** | | | | **LFT Test against Nested** | |
|  |  |  |  |  | **Participants** | **Items** | **AIC** | **BIC** | **LL** | **df** | **Df** | **X^2^** |
|  |  |  |  |  |  |  |  |  |  |  |  |  |
| RE only |  | - | - |  |  |  |  |  |  |  |  |  |
| RE only |  | - | - |  | Intercept | Intercept + Slope | 1056277 | 1056395 | -528129 | 8 |  |  |
|  |  |  |  |  |  |  |  |  |  |  |  |  |
| FE main effects | Main effects 1 | - | Trial Order + Semantic Rating + Pitch + Noise |  | Intercept | Intercept + Slope | 1056164 | 1056274 | -528070 | 11 | 3 | 118.233 |
| FE two-way interactions | Model 2 | Model 1 | Trial Order + Semantic Rating + (Pitch x Noise) |  | Intercept | Intercept + Slope | 1056160 | 1056288 | -528066 | 13 | 2 | 8.184 |

| **Supplement Table 2. Model comparison and the model building process for the effect of dynamic pitch/task on peak pupil dilation: quiet only** | | | | | | | | | | | | |
| --- | --- | --- | --- | --- | --- | --- | --- | --- | --- | --- | --- | --- |
| **Sampling Units** | | **N total observations = 76163**  **N Subjects = 22**  **N items = 144** | | | | | | | | | | |
|  | | | | | | | | | | | | |
| **Model specification** | **Model name** | **Nested/simpler model** | **Fixed Effects Added** |  | **Random Effects** | | **Model Fit** | | | | **LFT Test against Nested** | |
|  |  |  |  |  | **Participants** | **Items** | **AIC** | **BIC** | **LL** | **df** | **Df** | **X^2^** |
|  |  |  |  |  |  |  |  |  |  |  |  |  |
| RE only |  | - | - |  |  |  |  |  |  |  |  |  |
| RE only |  | - | - |  | Intercept+ Slope | Intercept | 1184628 | 1184711 | -592305 | 8 |  |  |
|  |  |  |  |  |  |  |  |  |  |  |  |  |
| FE main effects | Main effects 1 | - | Trial Order + Semantic Rating + Pitch + Task |  | Intercept+ Slope | Intercept | 1184541 | 1184652 | -592259 | 11 | 3 | 92.725 |
| FE two-way interactions | Model 2 | Model 1 | Trial Order + Semantic Rating + (Pitch x Task) |  | Intercept+ Slope | Intercept | 1184532 | 184661 | -592252 | 13 | 2 | 12.977 |
